# Water Transport through Cracked Concrete Structures—Effect of Mixture Proportion on Separating Crack Geometry and Permeability

**DOI:** 10.3390/ma15175807

**Published:** 2022-08-23

**Authors:** Lena Mengel, Hans-Werner Krauss, Dirk Lowke

**Affiliations:** Institute of Building Materials, Concrete Construction and Fire Safety, Technische Universität Braunschweig, Beethovenstraße 52, 38106 Braunschweig, Germany

**Keywords:** water permeability, separating cracks, crack geometry, concrete composition

## Abstract

The increase in fluid transport due to separating cracks can lead to significant deterioration in the durability of reinforced concrete structures. Besides reinforcement and stress state, concrete mixture proportion has a significant effect on crack geometry. In this study, we investigated concrete mixtures with different aggregate size and shape, aggregate gradation, cement type and water-to-cement ratio with regard to crack geometry and resulting water permeation. Besides surface-crack width and length, we determined inner-crack width variation over depth and tortuosity by X-ray micro-computed tomography. Furthermore, we conducted permeation tests for each specimen. Among the mixture components tested, aggregates have the strongest effect on crack geometry and flow rate. Increasing aggregate size results in increasing tortuosity and decreasing flow rate. Furthermore, the replacement of round with angular aggregates results in slightly higher flow rates for a given crack width.

## 1. Introduction

Permeability of concrete is a key factor with regard to serviceability and durability, as it highly determines moisture ingress and transport of chlorides or other corrosion-inducing substances [1,2,3,4]. In many cases, permeability is determined for uncracked concrete samples [5,6]. However, since the presence of cracks increases permeability up to several orders of magnitude [7,8,9,10,11], tests on cracked concrete specimens could give more realistic results with regard to durability of real structures [12]. However, a distinction must be made between different crack types, i.e., micro-cracks, bending cracks and separating cracks. Micro-cracks are unavoidable in concrete. But as they have a width of only up to 50 µm, they have no significant effect on water permeability. Bending cracks do not separate a concrete member in its entirety, but usually only extend as deep as the first layer of reinforcement. Separating cracks in this paper are cracks that pass completely through a concrete member or sample and thus separate it. This paper focuses on separating cracks, as they lead to an increase of permeability orders of magnitudes higher than the other crack types, which is in particular relevant for all water-resistant constructions, such as rainwater retention basins or biogas plants. In the past, a number of research activities have been conducted on the permeability of cracked concrete. For example, the effect of surface-crack width [13,14,15,16,17], permeating fluid [18,19] or different concrete strengths and reinforcement ratios [13,20] on permeability have been investigated. Recently, self-healing of concrete has been in the focus of research [12,21,22,23,24,25,26], including targeted effecting of self-healing [23,27,28,29,30]. However, self-healing is not in the focus of this work.

**Figure 1:**
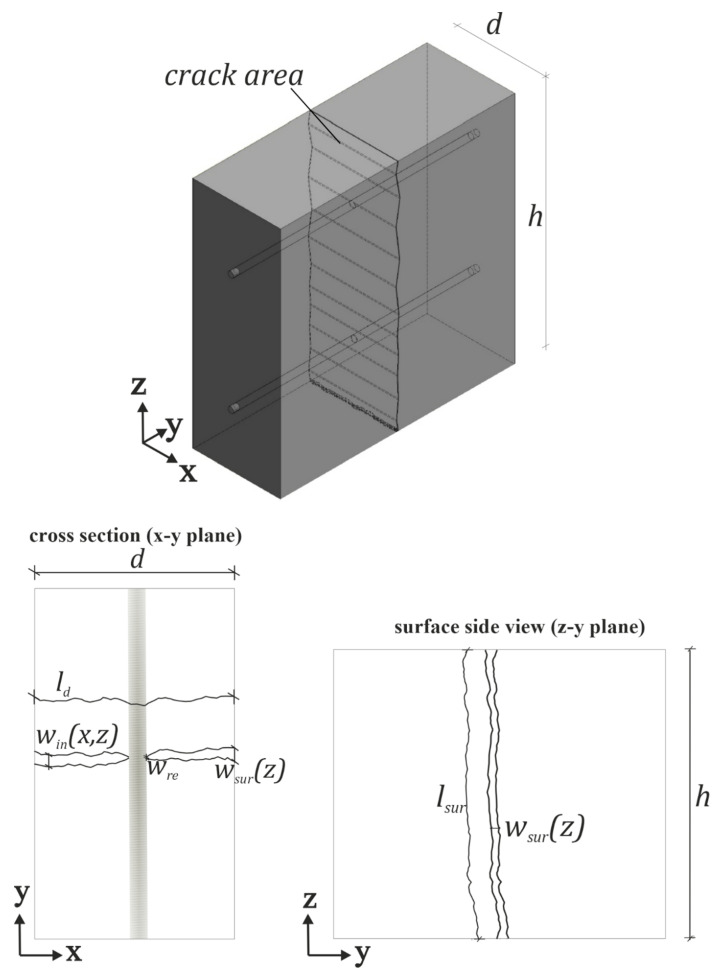
Schematic illustration of a separating crack in reinforced concrete.

In Figure 1, different crack geometry parameters at the concrete meso scale are illustrated. The analyzed scale is in the range of 0.05 to 50 mm. The crack geometry is most frequently described by the values surface-crack width *w_sur_(z)* and surface-crack length *l_sur_*. However, it should be noted here that in the individual studies the parameter crack width is used ambiguously. Often, crack width corresponds to the measured crack mouth opening at the surface *w_sur_(z)* (see Figure 1), but in addition, the calculation of crack width based on measured slip of a steel rebar relative to reference points on the concrete surface has been reported [31,32,33,34]. Thus, resulting crack width values are highly dependent on the measuring method and should therefore always be evaluated in the context of the exact test method used. A more precise description of the crack geometry on meso level is possible using the tortuosity τ, and variations in inner-crack width *w_in_(x,z)* over element thickness *d* and height *h*. Tortuosity is an intrinsic material property depicting the ratio of the effective transport path *l_d_* and the direct length *d*. However, different definitions of this ratio are existing. One definition is *l_d_/d* and another is the square or reciprocal form of this ratio [35,36]. In the case of uncracked porous materials, further definitions exist, which take the porosity or nominal aggregate size into account [37,38].

The crack width *w_in_(x,z)* of a specimen varies significantly, with the most important influencing factors being stress state in the cross-section, reinforcement and concrete mixture proportion. Prior research regarding the latter suggests that crack width and tortuosity in concrete are affected by different mixture components. It was shown that flow rate decreases with increasing maximum grain size [39,40], which could be caused by an increase in tortuosity and/or a smaller crack width ratio, i.e., the ratio of inner-crack width to surface-crack width of the crack width with increasing maximum grain size. Furthermore, an effect of the gradation of the aggregates on permeability was found by Meichsner [39]. He noticed a worse self-healing behavior with well-graded concretes. The shortest self-healing time was determined for a concrete with an almost monomodal grain size distribution of 2–4 mm. However, comprehensive knowledge about the effect of different concrete mixture parameters on crack geometry and permeability is not yet available.

In addition to crack width variations over depth *w_in_(x)* and cross-section height *w_in_(z)* [10,33,41], a clear difference can also be measured between surface-crack width *w_sur_* and crack width at the reinforcement *w_re_* [30,42,43,44]. The latter relationship is already considered in models proposed by, e.g., [45,46,47]. However, crack-width variation over depth can also have a decisive effect on permeability, as it is the bottleneck for the permeating fluid. Therefore, the consideration of more than the parameters surface-crack width and length is necessary with regard to the prediction of water permeability. Though some approaches exist to predict *w_in_(x)* with regard to reinforcement, cross-sectional design and mechanical properties of concrete [45,46,47], the implementation of the crack geometry into permeation models is a largely open issue.

At present, several permeation models assume cracks as smooth parallel plates and only consider the surface-crack width *w_sur_(z)* and surface-crack length *l_sur_* of the crack geometry. Usually, the theory of laminar flow of incompressible Newtonian fluids through parallel plates is used for permeation models, e.g., Poiseuille’s law [48], Equation (1):

q = *ξ l_sur_* w^3^_sur_ Δ*p*/(12 *η d*), (1)

where q is the flow rate, *l_sur_* is the surface length of the crack, *w_sur_* is the surface-crack width, Δ*p* is the pressure gradient, *η* is the dynamic viscosity of the fluid and *d* is the thickness of the element. The empirical correction factor *ξ* has been introduced to consider the effect of crack length along the thickness of the cross-section (*l_d_*) and variations in crack width over depth (*w(x)*, *w_re_*) as well as the crack surface roughness. The *ξ* values are reported in a wide range from 0.01 to 0.4. [26,33,49]. This is due to varying concrete mixtures, reinforcement types, reinforcement ratios, sample geometries and boundary conditions used.

To investigate the geometry crack parameters, e.g., tortuosity or *w_in_(x,z)*, complex measurement methods are necessary. In addition to test methods, which need destructive sample preparation such as sawing, non-destructive methods are more recently available, such as X-ray micro-computed tomography (µCT). With the development of this method, more comprehensive crack investigation became possible. [50,51,52,53] The µCT has been used for characterization of crack tortuosity and crack width ratio CWR [41], monitoring of fracture processes [52] or of water ingress [54]. One advantage of this method is, for example, the possibility to carry out non-destructive measurements.

In this paper, we focus on the effect of different concrete mixture parameters on crack tortuosity and crack-width variation over depth. Furthermore, we relate the inner-crack width to the surface-crack width as well as the flow rate through the crack. We present results on plain concrete. Based on these results, we evaluate and discuss the effect of mix composition on crack geometry and permeability.

## 2. Materials and Methods

### 2.1. Concrete Mixture Proportion

Concretes and mortars with different aggregate size and shape, aggregate gradation, water-to-cement ratio and cement type were tested. Gravel was used for round aggregates mixtures. For mixtures with angular aggregates, the 2–8 mm grain fractions were replaced with crushed basalt. The maximum aggregate size was 2, 4, 8, and 16 mm, respectively. Fine and coarse sieve curves were investigated (AB 8 and BC 8 according to DIN 1045-2 [55]). Two cements were tested, varying ordinary Portland cement and blast furnace slag cement. Furthermore, water-to-cement ratios of 0.5 and 0.6 were investigated. Table 1 shows the mixture compositions. Each mixture is named according to the following scheme. The first letter indicates the type of aggregate used. R represents round, A angular. The following number identifies the maximum aggregate size, varying from 2 to 16 mm. The water-to-cement ratio (*w*/*c*) used is specified by 0.5 or 0.6 at the next position, followed by OPC or BFSC for ordinary Portland cement or blast furnace slag cement. The aggregate gradation is then specified with coarse or fine for the concrete mixtures.

**Figure 2:**
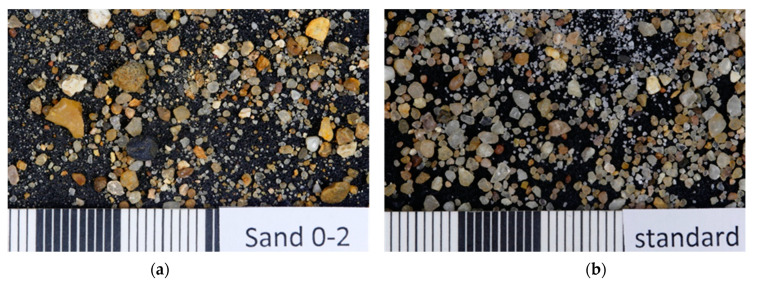
(**a**) Sand (0–2 mm) used for the mixture with 4, 8 and 16 mm maximum aggregate size, (**b**) Standard sand according to DIN EN 196 used for the mixture with 2 mm maximum aggregate size (R2-0.5-OPC and R2-0.6-OPC).

In contrast to the 4, 8 and 16 mm mixtures, for which local sand was used, the mixtures with a maximum aggregate size of 2 mm (R2-0.5-OPC, R2-0.6-OPC) were made with standard sand according to DIN EN 196 (0–2 mm grain size, [56]). One of each sand type is shown in Figure 2.

### 2.2. Preparation and Cracking of Samples

**Figure 3:**
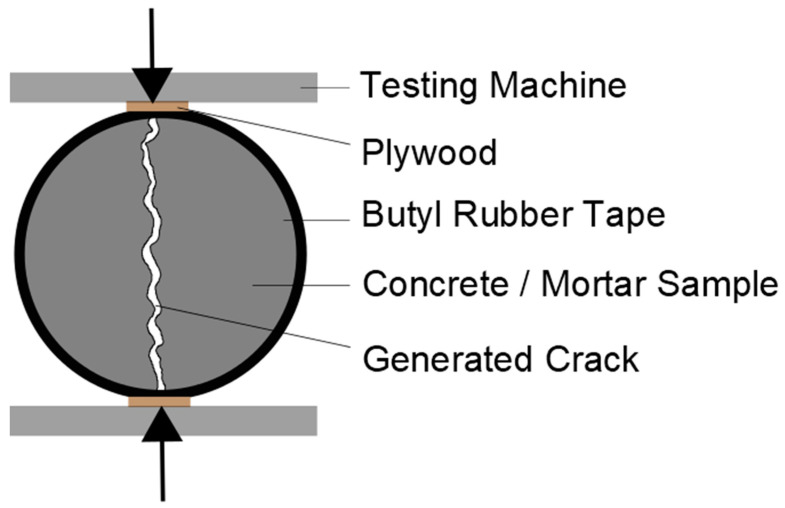
Scheme of the crack generation test setup.

The samples were cast in cylindrical PE-molds with a diameter of 50 mm and were stored in 20 °C and 65% relative humidity for 28 days. The casted concrete cylinders were demolded after 1 day and cut to a length of 50 mm. The cylinders were wrapped with a butyl rubber tape to avoid sudden failure during crack generation and to seal the lateral surfaces for permeation tests. Cracks were generated at the age of 7 days after casting by a displacement controlled splitting tension test at a velocity of 0.025 mm/s. As a result, after unloading, surface cracks with a width of 0.05–0.45 mm were “randomly” generated. The test setup of crack generation can be seen in Figure 3.

### 2.3. Permeation Tests

**Figure 4:**
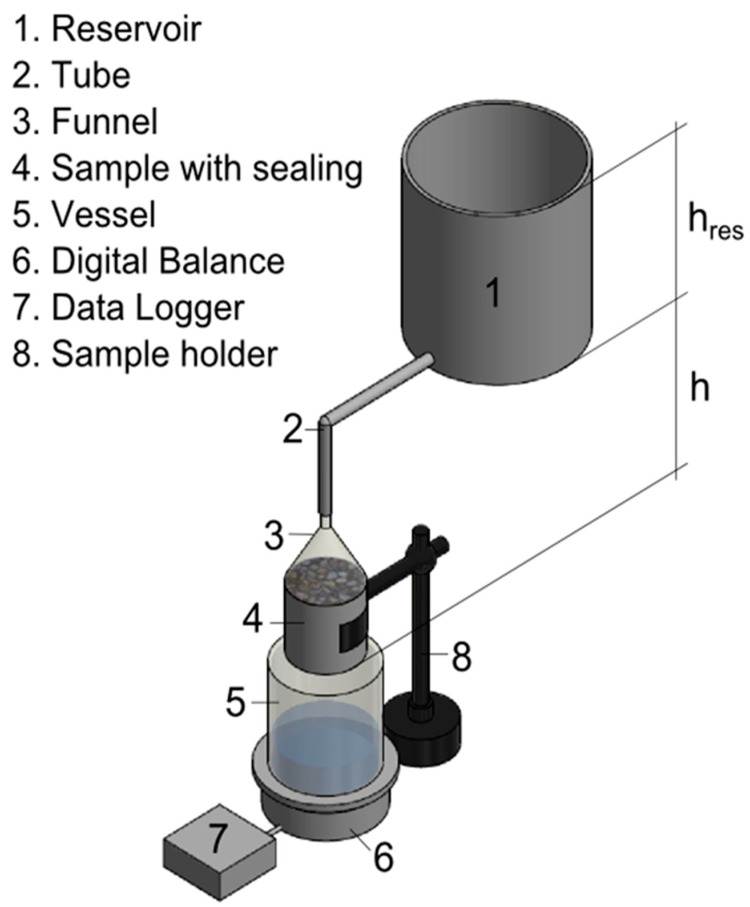
Scheme of the permeation test setup.

Permeation tests were carried out at a sample age of 28 days. Figure 4 shows the test setup. To apply a constant water pressure, a funnel connected to the water reservoir was glued to the sample surface. In conjunction with the butyl rubber tape a transport of water was ensured through the crack and concrete matrix. Water pressure height (*h_res_ + h*) was kept constant at a height of 55 cm, i.e., 5.4 kPa, which corresponds to a pressure gradient of 11 m/m (height of the fluid relative to the thickness of the sample) for all tests, with atmospheric pressure at the discharge outlet. The specified water level in the reservoir was visually checked. Water level changes were immediately compensated by refilling the reservoir. The permeating liquid was collected in a vessel on a digital balance with an accuracy of 0.1 g, with continuous data logging. As prior research has shown that self-sealing phenomena can reduce the permeability of a crack [19,25,26,57] within hours to weeks, the flow rate was determined after 20 min to prevent self-sealing effects.

### 2.4. Determination of Crack Geometry

The crack geometry is described based on the measured values surface-crack width *w_sur_(z)*, inner-crack width *w_in_(x,z)*, surface-crack length *l_sur_* and crack length *l_d_* in x-direction, i.e., in flow direction through the width of the cross-section, see Figure 1. These values were measured as polylines on pictures taken with the µCT. This approach enabled the characterization of these values without the need of fixing the crack. To exclude possible changes of crack geometry due to self-sealing effects, all samples were scanned prior to the permeation tests.

**Figure 5:**
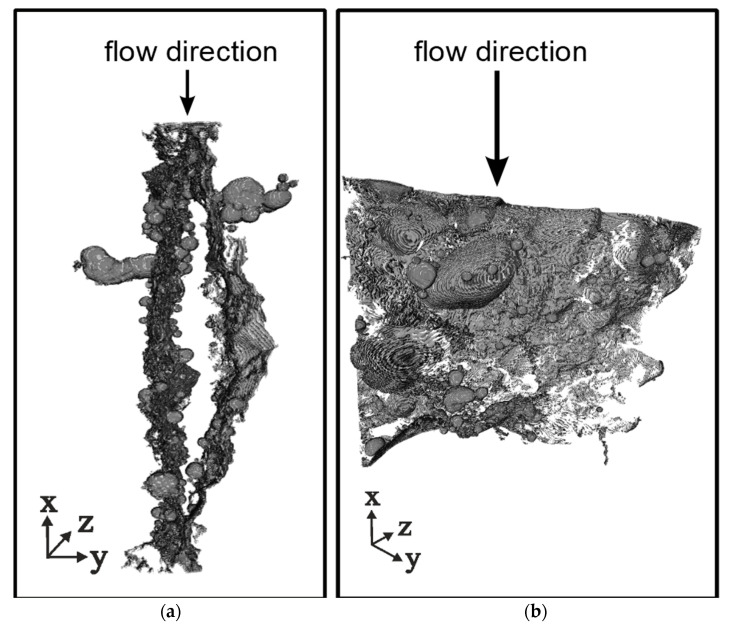
3-dimensional crack geometry processed with a Matlab script (voxel edge length: 58 µm) (**a**) Local branching into two cracks, (**b**) Areas with discontinuities.

In this research, the specimens were scanned with a GE phoenix|v tome|x s cone X-ray CT device (GE Sensing & Inspection Technologies GmbH, Hürth, Germany). Parameters for the CT scans were 160 kV tube voltage, 250 µA tube current and 333 ms exposure time with a 0.1 mm Cu-filter. The scans had a voxel edge length of 58 µm and contain 1000 pictures. The three-dimensional (3D) structure was reconstructed with phoenix datos|x2 (GE Sensing & Inspection Technologies GmbH, Wunstorf, Germany) and converted to RAW-files with VGStudio 2 (Volume Graphics GmbH, Heidelberg, Germany). RAW-files were imported into Matlab 2018a (The Mathworks, Inc., Natick, MA, USA) and processed with a custom script. The script is characterized by automatic threshold segmentation of the crack, based on the different absorption of X-ray radiation by phases air, hardened cement paste, aggregates and butyl rubber tape. With this procedure, air, concrete and butyl tape were defined, chosen and segmented. At a next step, a region of interest (concrete and included air) was further processed. Grey value images were binarized to black and white areas and a volume analysis was carried out. Thus, the crack space as the largest cluster was extracted. Exemplary results of a Matlab processing are shown in Figure 5, displaying the 3D crack geometry within the sample. Obvious characteristics are changes in crack length and width in x- and z-direction, tilts in the crack path and branching of the crack. In Figure 5a, the local branching into two cracks is visible, whereas in Figure 5b areas with discontinuities of the crack can be identified. Furthermore, failure along the ITZ between aggregates and cement paste can clearly be seen in Figure 5b. The gained 3D data are used for further geometry analysis to evaluate tortuosity and crack-width variation over depth and cross-section (see Section 3.2).

In addition to µCT measurements, the surface-crack width *w_sur_(z)* was determined using (a) crack-width ruler and (b) digital microscope Keyence VHX-2000 (Keyence Deutschland GmbH, Neu-Isenburg, Germany). Therefore, a total of six *w_sur_(z)* values were measured on the sample’s surface (three on the top and three on the bottom surface).

### 2.5. Parameters Characterizing the Crack Geometry

**Figure 6:**
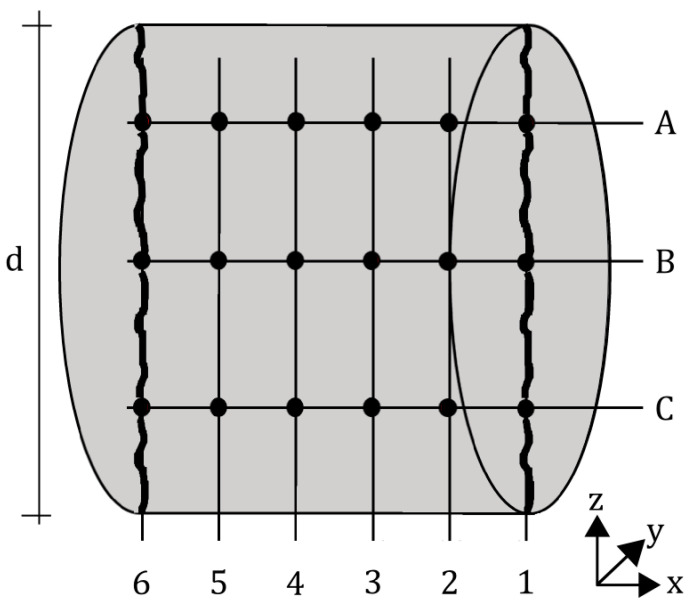
Position of measuring points within a sample.

For the characterization of the crack geometry, the following parameters were calculated: (a) the mean surface-crack width *w_sur,m_*, (b) the minimum mean inner-crack width *min. w_in,m_* of the y-z-planes 1–6 according to Figure 6, (c) the crack-width ratio CWR in x-direction and (d) the tortuosity τ in x-direction.

The y-z-planes are used to evaluate the crack-width ratio (CWR), which is the ratio of the minimum mean inner-crack width *min. w_in,m_(z)* of the six y-z-planes 1–6, to the mean surface-crack width *w_sur,m_*. For the purpose of identifying *min. w_in,m_*, a mean crack width *w_in,m_* for each of the six y-z-planes was calculated based on three measured values (A, B, and C) (Figure 6). The six values were compared, and the smallest of them is hereinafter referred to *min. w_in,m_*.

The definition of the minimum mean inner-crack width was based on the assumption that a smallest crack opening area in a sample exists that is decisive for the transport of water, similar to a bottleneck. Even if a widening of the crack takes place above or below (x-direction) this smallest area, a larger transport will still not occur. At the same time, from this point of view, the absolute minimum measured crack width in a sample cannot be decisive for the transport, since a larger crack width can be present along the crack path in z-direction. Therefore, the water would not flow through the absolute minimum crack width. As a result, the minimum crack area must be decisive for the flow of water, characterized here as minimum mean crack width in z-direction *min w_in,m_(z)*.

Furthermore, crack tortuosity τ in x-direction, i.e., the flow direction, was evaluated in the three x-y-planes A, B, C (Figure 6). It is defined as the ratio of crack length and cross-section width *l_d_/d*. For each mixture, one mean tortuosity value was determined. Therefore, the mean value of the three tortuosities in flow direction measured per sample was determined. These mean values then serve as the basic population for determining the tortuosity for a mixture.

## 3. Results and Discussion

### 3.1. Surface-Crack Width

**Figure 7:**
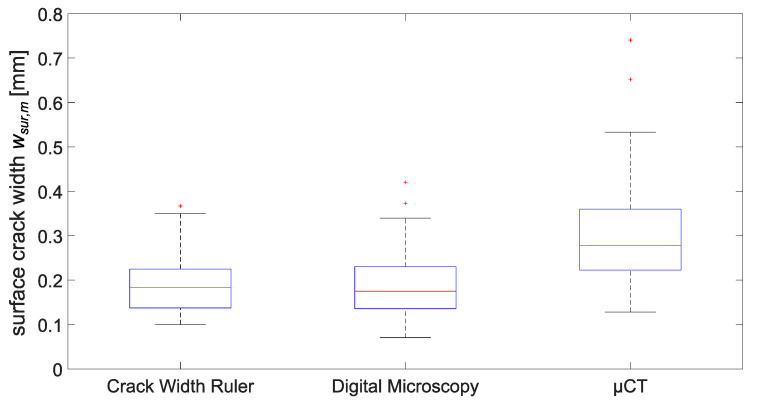
Comparison of surface-crack width results from different measurement methods (bottom and top edge of a box indicate the 25th and 75th percentiles, the band inside a box is the median, whiskers extend to the minimum and maximum data point within the 1.5 times interquartile range, a plus sign represents an outlier).

Three different measurement methods were used here to determine the mean surface-crack width *w_sur,m_*, i.e., crack-width ruler, digital microscope and µCT. Here, *w_sur,m_* is a mean value based on six measuring points (three on the top and three on the bottom surface, layer 1 and 6 (Figure 6). Comparing the results in Figure 7, µCT data give higher *w_sur,m_* values than the crack-width ruler and digital microscope, with the crack-width ruler and digital microscope being in the same range.

Higher µCT values may be explained by the characteristics of the µCT measurement procedure. In this method, one scan contains 1000 images of the y-z plane. Accordingly, the crack width is defined here as the extension in the y-direction. However, the specimens cannot be mounted absolutely vertically in the µCT, i.e., the end face of the cylinder is not absolutely parallel to the y-z plane. Therefore, the measured µCT crack width has not only a horizontal but also a small vertical component resulting in larger values.

These results emphasize once again that the measuring method plays a decisive role in determining the crack width and must therefore be specified for reasons of comparability. Within the scope of our investigations, we used the digital microscope as an exact quantification of the surface-crack width *w_sur,m_(microscope)*. For the calculation of the relative parameter CWR, we used the value from µCT measurements, i.e., surface-crack width *w_sur,m_(µCT)*, as this is related to the inner-crack width that was also determined in the µCT.

### 3.2. Tortuosity and Crack Width Ratio

#### 3.2.1. Tortuosity

The calculation of tortuosity is based on at least 9, normally 18 and a maximum of 33 measured tortuosities. Table 2 gives the calculated tortuosity parameters for the investigated concrete and mortar mixtures in flow-direction (x-direction).

**Figure 8:**
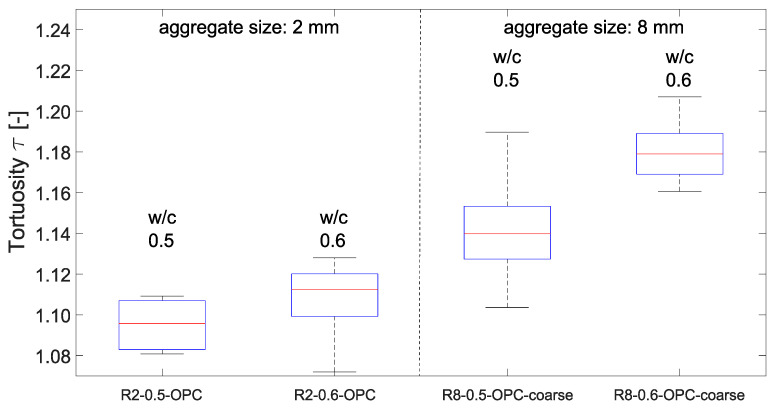
Effect of water-to-cement ratio on tortuosity for mixtures with 2 mm and 8 mm maximum aggregate size (bottom and top edge of a box indicate the 25th and 75th percentiles, the band inside a box is the median, whiskers extend to the minimum and maximum data point within the 1.5 times interquartile range).

In Figure 8 the tortuosities for the different *w*/*c*-ratios are displayed for the mixtures with 2 mm and 8 mm maximum aggregate size. For mixtures with *d_max_* = 2 mm the median tortuosity increases by 0.01 from *w*/*c* = 0.5 to 0.6, whereas by 0.04 for *d_max_* = 8 mm. The higher tortuosities for *d_max_* = 8 mm compared to *d_max_* = 2 mm are likely due to the size of maximum aggregate diameter. It has to be mentioned that the aggregate’s volume fraction was, in order to produce workable mixtures with same sieve curves, not constant for all mixes. The volume increases slightly by 2% and 3% from *w*/*c*-ratio 0.5 to 0.6 for a grain size of 2 and 8 mm, respectively.

**Figure 9:**
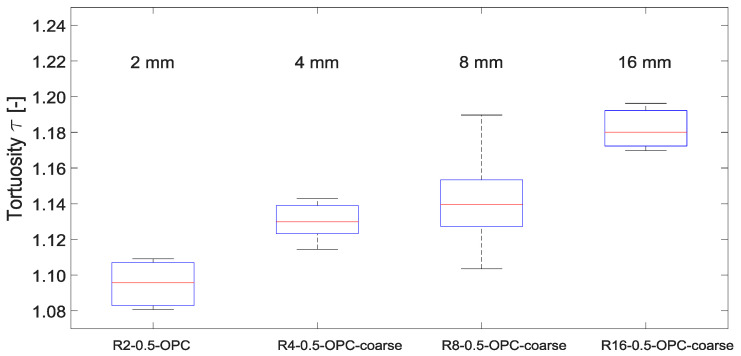
Effect of the maximum aggregate size on tortuosity (bottom and top edge of a box indicate the 25th and 75th percentiles, the band inside a box is the median, whiskers extend to the minimum and maximum data point within the 1.5 times interquartile range).

A clear correlation between maximum aggregate size *d_max_* and tortuosity is evident from further results shown in Figure 9. The median increases from 1.10 for *d_max_* = 2 mm to 1.18 for *d_max_* = 16 mm. Note: While the difference in aggregate volume between grain size 2 mm and 4 mm is 3% and between 4 mm and 8 mm 9%, the aggregate volume fraction of 8 mm and 16 mm is constant.

The clear effect of aggregate size can be explained by the failure mechanism of normal-strength concrete, according to which the crack usually occurs along the interfacial transition zone. Thus, the larger the aggregates’ diameter, the larger the circumference is and thus the crack path.

No significant effect of cement type on tortuosity could be found for the two cement types used for grain size 16 mm (R16-0.5-OPC-coarse vs. R16-0.5-BFSC-coarse), compare Table 2. The median of the mixture with blast furnace slag cement was higher, while the 25% quantile and minimum were equal or rather lower than for the mixture with ordinary Portland cement. In contrast, 75% quantile and maximum were significantly higher. Note that although no correlation could be measured here, it is still possible that a change of cement type leads to a change in the roughness of the hardened cement paste at micro scale.

Further evaluations presented no correlation between the investigated aggregate gradation and tortuosity, e.g., median, quantile and extreme values are in similar range. Only maximum and the 25% quantile of the fine sieve curve (R8-0.5-OPC-fine) were slightly lower than for the coarse sieve curve (R8-0.5-OPC-coarse) (Table 2).

**Figure 10:**
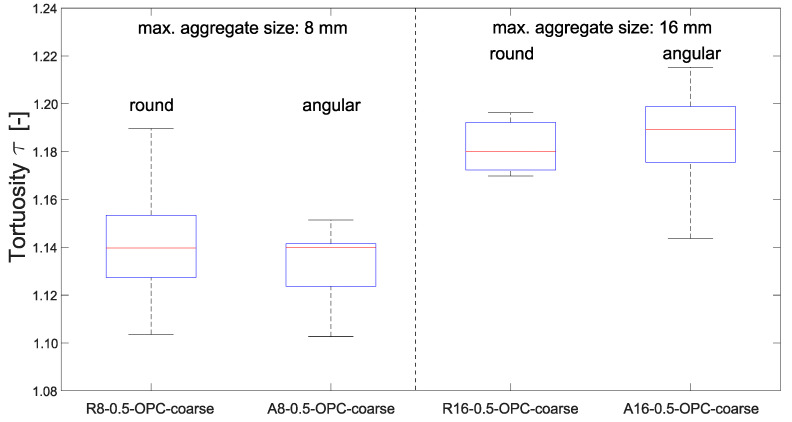
Effect of the aggregate shape on tortuosity (bottom and top edge of a box indicate the 25th and 75th percentiles, the band inside a box is the median, whiskers extend to the minimum and maximum data point within the 1.5 times interquartile range).

Furthermore, a comparison between angular and round aggregates showed no significant effect of aggregate shape on tortuosity (Figure 10). Medians of the angular aggregates were slightly higher, but 25% and 75% quartiles as well as the minimum and maximum values did not reveal a significant difference. Initially, a higher tortuosity of the angular aggregates was expected. However, the investigation of the crack geometry in µCT displayed that in specimens with angular shaped aggregates, the crack more often passes directly through the aggregates. In round aggregate specimens, on the other hand, the crack usually passes around the aggregates, which could be a reason for the almost identical tortuosities of angular and round-shaped aggregates.

#### 3.2.2. Crack-Width Ratio

For a precise determination of the effect of crack width on permeability, not only the surface-crack width *w_sur_* but also the internal crack width *w_in_* must be taken into account. Therefore, a crack-width ratio CWR was determined. The CWR describes the ratio of the minimum mean inner-crack width to the mean surface-crack width, CWR = *min. w_in,m_/w_sur,m_*. Consequently, a small CWR represents a significantly smaller mean crack width inside than on the specimen’s surface. For the calculation of the CWR, the surface-crack width determined in the µCT was used.

**Figure 11:**
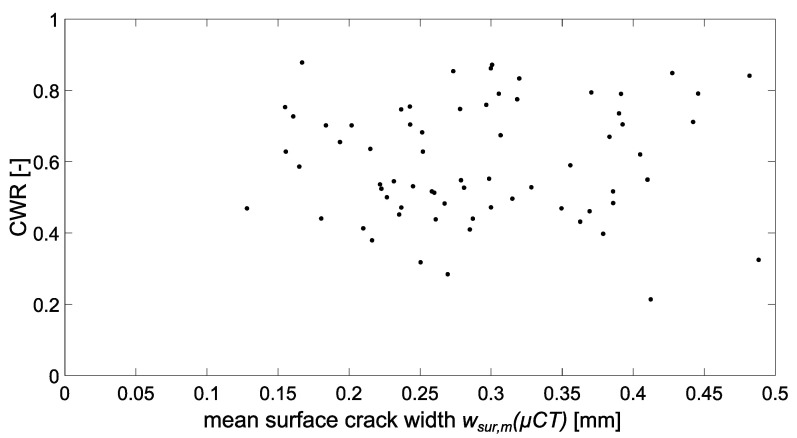
Crack-width ratio CWR in relation to the mean surface-crack width *w_sur,m_*.

**Figure 12:**
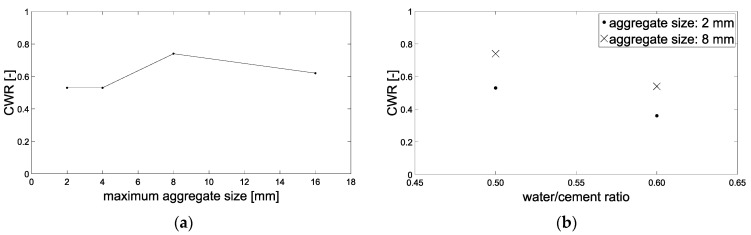
Crack-width ratio CWR: (**a**) Effect of maximum aggregate size, (**b**) Effect of *w*/*c*-ratio.

**Figure 13:**
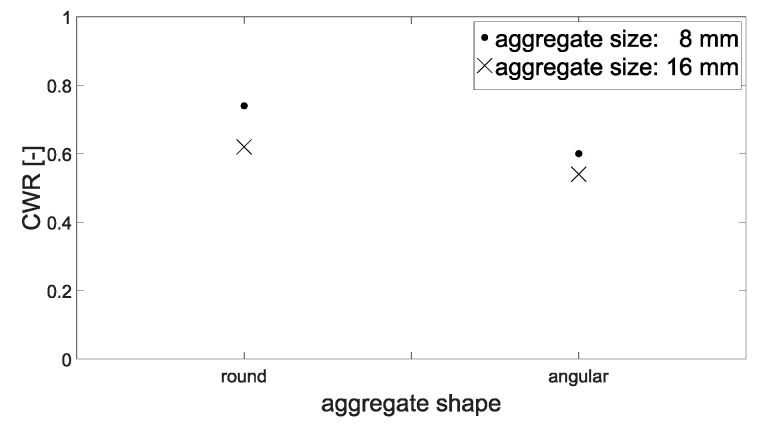
Crack-width ratio CWR: effect of aggregate shape.

Figure 11 depicts the distribution of CWR values in relation to the mean surface-crack width for the mixtures investigated. According to this, the crack width inside the samples is only approx. 30 to 90% of the crack width at the surface. This significantly smaller inner-crack width leads to considerably lower flow rate than would be expected on the basis of surface-crack width. However, there is no direct correlation between surface- and inner-crack width. Thus, the surface-crack width has only limited validity for characterizing the crack narrowing in the inner structure. Here, the concrete composition also seems to have a significant effect. Therefore, an average CWR was determined for each mixture and compared with the mean value of the other mixes-, (Figure 12 and Figure 13).

According to Figure 12a, a maximum aggregate size of 8 mm results in the highest CWR, while the mixtures with 2 and 4 mm have lower CWR. One explanation for this could be that the higher tortuosity at larger aggregates (compare Figure 9) can result in the cracks generated not deforming back as much after cracking and unloading as is the case with smaller maximum aggregate size.

In Figure 12b, the comparison between a water-to-cement ratio of 0.5 and 0.6 can be seen. The higher *w*/*c* value here was accompanied by smaller CWR. The difference was about 20%. Finally, Figure 13 gives the difference between round and angular aggregate. Here, the angular aggregates led to a lower CWR, i.e., narrower inner cracks compared to the surface-crack width, with the difference being about 12%.

### 3.3. Flow Rate

**Figure 14:**
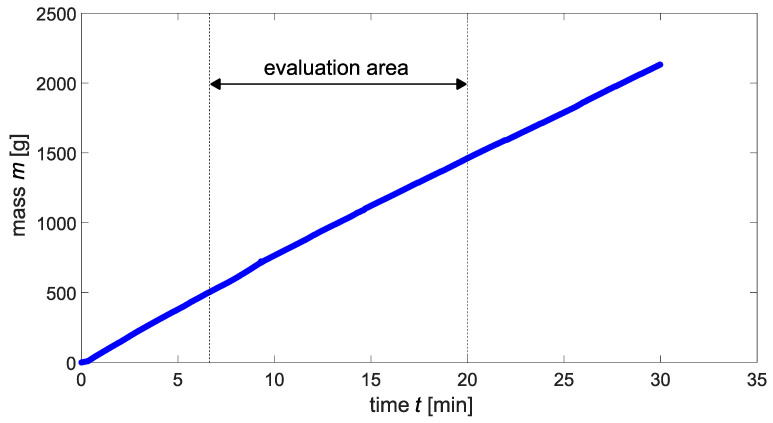
Exemplary result of a permeation experiment.

An exemplary result of a permeation experiment is shown in Figure 14. In some experiments the initial mass flow was nonlinear, so this range was not taken into account for the determination of the flow rate. Usually, a linear mass transport was measurable at the latest from a test duration of 7 min, which is why a standard evaluation range between 7 and 20 min was chosen.

**Figure 15:**
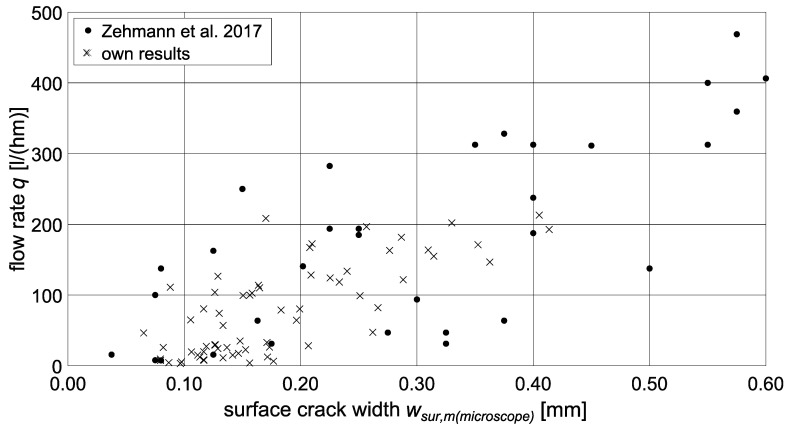
Flow rate q in dependence of mean surface-crack width *w_sur,m_*. Comparison of experimentally determined flow rate values to literature [58].

The comparison of the measured flow rates in dependence of the mean surface-crack width *w_sur,m_* with data from the literature [58] showed comparable trends as displayed in Figure 15. The scatter of the results was also in a similar range as can be seen from the coefficients of determination for a polynomial fit. Both sealing method and test setup were used in this study to determine the flow rate are therefore suitable for flow-rate investigations. Basically, surface-crack width correlates with flow rate, the latter increasing with increasing crack width. However, as discussed before, regarding a prediction based on the cubic law, the use of the surface-crack width leads to a significant overestimation of the permeability (compare *ξ* in Equation (1)). The reason for this is the considerably smaller inner-crack width as shown in Section 3.2.2, Figure 11. Due to the bottleneck effect, the minimum inner-crack width must therefore be the determining factor for the flow rate. Hence, the inner-crack width is used as reference value for the evaluation of the flow rate results in the following section.

**Figure 16:**
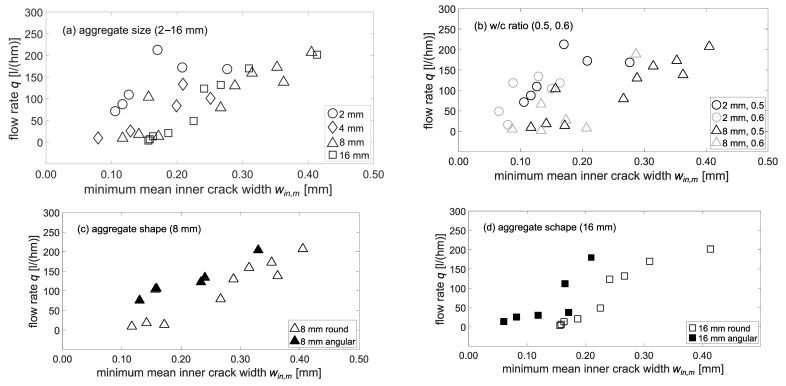
Effect of minimum mean inner-crack width *min. mean w_in,m_* on flow rate q in dependence of (**a**) Aggregate size (2–16 mm, round, *w*/*c*: 0.5); (**b**) v-ratio (round), (**c**) Aggregate shape 8 mm (*w*/*c*: 0.5), (**d**) Aggregate shape 16 mm (*w*/*c*: 0.5). All concrete mixtures shown here were made with a coarse sieve curve and ordinary Portland cement; (µCT voxel edge length: 58.2 µm).

The following Figure 16a–d illustrates the effect of the inner-crack width on the flow rate in dependence of the concrete mixture variations investigated. As the characteristic value, the minimum mean inner-crack width *min. w_in,m_* of the six y-z-planes according to Figure 6 was used to capture the highest flow resistance (bottleneck effect) in the x-direction, i.e., the flow direction.

First of all, it can be noted that analogous to the mean surface-crack width *w_sur,m_*, the flow rate increases with increasing minimum mean inner-crack width *min. w_in,m_*. Figure 16a shows the effect of aggregate size on flow rate. Here, a trend towards lower flow rates for a given crack width with increasing maximum aggregate size can be observed. This trend is unaffected by the *w*/*c* ratio, Figure 16b. These observations correspond to the higher tortuosity values measured with higher *d_max_*, compare Figure 9. Due to increasing changes in direction of the flow of water in the crack with increasing tortuosity, the flow resistance in the crack increases, i.e., the flow rate *q* decreases. The measured decrease in flow rates with higher *d_max_* is also in agreement with findings of Tuskamoto [2].

When comparing round and angular aggregates, the angular aggregates seem to correspond to slightly higher flow rates for a given crack width-, (Figure 16a,b). Consideration of the tortuosity of the different concrete mixes shows no significant differences, compare Figure 10. Thus, tortuosity does not seem to be responsible for differences in flow rate. Further influencing factors could be surface properties and micro-roughness of the crack walls, which, however, were not considered in our investigations.

## 4. Conclusions

The aim of this study was to investigate the crack geometry of separating cracks at meso level as well as the water transport through cracked concrete structures. The focus was particularly on the effect of concrete composition on crack geometry parameters and the resulting flow rate. Therefore, we examined plain cracked concrete specimens using µCT to determine the surface-crack width *w_sur,m_*, the crack-width deviation over depth and height *w_in_(x,z)*, minimum mean inner-crack width *min. w_in,m_* and tortuosity τ. We also determined the surface-crack width *w_sur,m_* by digital microscope and crack-width ruler to investigate the comparability of the three measuring methods. In order to investigate the crack geometry with regard to its effect on the flow rate, we carried out permeation tests. The findings can be summarized as follows:

- A literature review revealed that the crack width is an ambiguous definition. It can be determined with different measuring methods and partly at locations deviating from the direct sample surface. Therefore, for the purpose of comparability, the measuring method and the measuring location must be reported when specifying the crack width;
- The use of microcomputed tomography (µCT) has great potential for the non-destructive determination of crack geometry. In addition to simple visualization, further processing of the µCT data is possible to determine the crack geometry inside the sample quantitatively. However, difficulties in the analysis of µCT scans involve areas of similar grey levels, as it is not straightforward to distinguish between different materials;
- µCT data give higher surface-crack widths *w_sur,m_* than the crack-width ruler and digital microscope, with the crack-width ruler and digital microscope being in the same range. If only the surface-crack width is necessary, a digital microscope is recommended;
- The evaluation of µCT scans shows a clear difference between the mean surface-crack width and the inner-crack width *w_in_(x,z)* or minimal mean inner-crack width *min. w_in,m_*. In most cases, inner-crack widths are significantly smaller than mean surface-crack width (CWR < 1). This significantly smaller inner-crack width is likely to result in a significantly lower permeability than would be expected based on the surface-crack width. The effect of the varying crack geometry inside a concrete member is therefore of great importance for prediction models;
- For natural round aggregates, an increasing aggregate size results in an increase of tortuosity and a decrease of permeability for a given crack width. With increasing tortuosity, the flow length and the changes in flow direction increase, which results in reduced permeability;
- The use of angular aggregate tends to result in smaller crack-width ratios, i.e., a stronger narrowing of the cracks inside the component. However, at a given inner-crack width, concrete mixes with angular aggregates show slightly higher flow rates than mixes with round aggregates. The tortuosities of both aggregate shapes are in a similar range;
- For *w*/*c*-ratio, cement type and aggregate gradation, we could not determine any clear effects on tortuosity and flow rate. Although a higher *w*/*c*-ratio appears to increase tortuosity and to reduce CWR, no effect was seen in flow-rate experiments;
- The aforementioned findings regarding the effects of various mixture parameters can be used to refine or redevelop predictive models for the flow through separating cracks. In particular the consideration of tortuosity and crack-width ratio could be beneficial;
- Furthermore, the results can be used for the development of an optimal concrete mixture. The results imply that the use of aggregates with a large maximum grain size can reduce the crack permeability.

## Figures and Tables

**Table 1:** Investigated concrete mixtures with overview of varied mixture components (abbreviations: R = round aggregates, A = angular aggregates, d_max_ = maximum aggregate size, Agg. = Aggregate, *w*/*c* = water-to-cement ratio, OPC = Ordinary Portland Cement, BFSC = Blast Furnace Slag Cement, Adm. = Admixture, SP = Superplasticizer).

Mix	Cement [kg/m^3^]	Water [kg/m^3^]	Agg. [kg/m^3^]	Agg. [vol.%]	d_max_ [mm]	Sieve Curve	*w*/*c*-Ratio	Cement Type	Adm.
R2-0.5-OPC	586	293	1758/R	51.8	2	standard sand	0.5	OPC	-
R2-0.6-OPC	586	352	1758/R	54.9	2	standard sand	0.6	OPC	-
R4-0.5-OPC-coarse	476	238	1412/R	59.4	4	coarse	0.5	OPC	-
R8-0.5-OPC-coarse	370	185	1613/R	68.1	8	coarse	0.5	OPC	-
R8-0.5-BFSC-coarse	434	217	1488/R	68.1	8	coarse	0.5	BFC	-
R8-0.6-OPC-coarse	305	183	1668/R	70.4	8	coarse	0.6	OPC	-
A8-0.5-OPC-coarse	396	195	1995/C	65.9	8	coarse	0.5	OPC	SP
R8-0.5-OPC-fine	390	195	1643/R	66.42	8	fine	0.5	OPC	-
R16-0.5-OPC-coarse	370	185	1721/R	68.07	16	coarse	0.5	OPC	-
R16-0.5-BFSC-corase	370	185	1721/R	62.7	16	coarse	0.5	BFC	-
A16-0.5-OPC-coarse	370	185	1990/C	68.1	16	coarse	0.5	OPC	SP

**Table 2:** Tortuosity τ in flow-direction (x-direction).

Mix Notation	Median	25th Percentile	75th Percentile	Minimum	Maximum
R2-0.5-OPC	1.10	1.08	1.11	1.08	1.11
R2-0.6-OPC	1.11	1.10	1.12	1.07	1.13
R4-0.5-OPC-coarse	1.13	1.12	1.14	1.11	1.14
R8-0.5-OPC-coarse	1.14	1.13	1.15	1.10	1.19
R8-0.5-BFSC-coarse	1.15	1.14	1.15	1.13	1.16
R8-0.6-OPC-coarse	1.18	1.17	1.19	1.16	1.21
A8-0.5-OPC-coarse	1.14	1.12	1.14	1.10	1.15
R8-0.5-OPC-fine	1.14	1.12	1.15	1.10	1.16
R16-0.5-OPC-coarse	1.18	1.17	1.19	1.17	1.20
R16-0.5-BFSC-coarse	1.19	1.17	1.27	1.16	1.37
A16-0.5-OPC-coarse	1.19	1.18	1.20	1.14	1.22

## Data Availability

Not applicable.
